# Exposure to PM2.5 via vascular endothelial growth factor relationship: Meta-analysis

**DOI:** 10.1371/journal.pone.0198813

**Published:** 2018-06-18

**Authors:** Yi Sun, Yao Wang, Shu Yuan, Jialing Wen, Weiyu Li, Liu Yang, Xiaoyan Huang, Yanmei Mo, Yingqi Zhao, Yuanming Lu

**Affiliations:** 1 Department of Toxicology, Guilin Medical University School of Public Health, Guilin, China; 2 The Library and Information Center, China Pharmaceutical University, Nanjing, China; 3 Guangdong Provincial Key Laboratory of Colorectal and Pelvic Floor Diseases. The Sixth Affiliated Hospital, Sun Yat-sen University, Guangzhou, China; 4 181st Hospital of People's Liberation Army of China, Guilin, China; Medical University Innsbruck, AUSTRIA

## Abstract

This study investigated the association of PM2.5 exposure with VEGF by conducting a systematic review of existing literature and performing a meta-analysis. We searched all the studies published in the Cochrane Library, PUBMED, Embase, China National Knowledge Infrastructure China National Knowledge Infrastructure, and WanFang Electronic Database before June 2017. Finally six studies were identified. It confirmed that the increase in VEGF (β = 1.23 pg/ml, 95% *CI*: 0.45, 2.01) was significantly associated with the PM2.5 mass concentration of 10 μg/m^3^. Studies from Canada showed that PM2.5 exposure statistically elevated the level of VEGF level that an increase of 1.20 pg/ml (95% *CI*: 0.88, 1.52) in VEGF was associated with per 10 μg/m^3^ increase in PM2.5 concentration. Other subgroup analyses indicated that the effects of PM2.5 exposure on VEGF differed per the in different exposure assessment methods, study designs, and study settings. It was concluded that elevated VEGF levels was significantly positive associated with PM2.5 exposure. Exposure assessment methods and study countries were the major sources of heterogeneity among studies.

## Introduction

Environmental particulate matter (PM) is defined as the microscopic solid or liquid matter suspended in atmospheric aerodynamic. PM2.5 refers to particulates less than 2.5 microns, also known as inhalable lung particles that causes negative impact on human health and atmospheric environmental quality. PM2.5 pollution in China has led to wide concern from all over the world. According to Chinese Environmental Monitoring Report, there was three large-scale regional haze pollution breaking out, including heavy pollution for 155 days and serious pollution for 31 days in 161 cities only from October 1 to October 24 in 2016. The epidemiological results have demonstrated that long-term exposure to PM2.5 promoted the increase of lung cancer incidence and played significant role in the rising of population mortality rate [1–4]. In addition, PM2.5 exposure was also proved to be a risk factor for cardiovascular disease [5], affecting vascular endothelial dysfunction [6], mediating vasodilation, accelerating the progression of atherosclerotic plaques [7].

More and more clinical trials have found that exposure to environmental fine particles increases neovascularization [8], and it is known that angiogenesis is essential for the occurrence and development of cardiovascular disease and tumors [9–11]. Angiogenesis and vascular remodeling lead to a variety of cardiovascular diseases such as myocardial ischemia, peripheral arterial disease, atherosclerosis and aortic aneurysm [12–14].Angiogenesis are thought to be a critical process of tumor growth and metastasis, and pathologic angiogenesis are a sign of cancer and various ischemic and inflammatory diseases [15–18].

VEGF is the most potent angiogenic growth factor known to date, capable of inducing endothelial cells proliferation and capillary formation, and is one of the major components taking part in vascular remodeling [19–20]. The increase in VEGF levels following PM and endotoxin exposure might be an acute systemic response to endothelial injury. The increased VEGF strongly promotes the proliferation of endocardial cells, significantly induced myocardial and endocardial damage [21]. Abnormal elevation of local VEGF expression plays an important role in the regulation of angiogenesis in the pathogenesis of heart disease and the angiogenesis of the tumor [22–23]. However, the results of literature on the correlation between PM2.5 exposure and VEGF level are inconsistent [24–29]. Therefore we conducted a meta-analysis of the existing literature to quantify the association between PM2.5 exposure and elevated VEGF levels, moreover to explore the role and the possible underlying molecular mechanisms of PM2.5 exposure induced VEGF expression in the process of cardiovascular disease and to evaluate the value of VEGF as a biomarker of cardiovascular diseases caused by PM2.5 exposure. In addition, a more recent study [30] demonstrated that episodic exposure to PM2.5 induced decrease of CEPCs. VEGF increased is thought to be, at least partially, responsible for the decreased number of CEPCs since it enhances homing and adhesion of the mobilized CEPCs to the damaged vascular endothelial sites, which may further reduce the number of CEPCs stored in the bone marrow thereby potentially exhausting the pool [31, 32].

Meta-analysis is the most common statistical technique used for quantitative and comprehensive analysis of results from two or more individual studies [33]. To more accurately estimate the association between PM2.5 exposure and VEGF elevation, we conducted a meta-analysis of all of the published studies. In this analysis, we systematically investigated previously published literatures on the topic, and used the meta-analysis model to quantitatively evaluate the effects of PM2.5 exposure on VEGF level. We further explored the influences of exposure measurement methods, study design, biomarker measurement sources, and count on the meta-estimation of PM2.5.

## Materials and methods

We searched the following database for studies published before June 2017: Cochrane Library, PUBMED, Embase, China National Knowledge Infrastructure, and Wanfang Electronic Database. Our search strategy used a combination of the following keywords for PM2.5: 'air pollution', 'particulate matter', 'ambient particulate matter', 'PM', 'PM2.5', 'PM10', 'airborne particulate matter', 'particulate air pollutants', 'black carbon', 'BC (black carbon)', as well as the following keywords for neovascularization: 'neovascularization', 'Angiogenesis, Pathologic', 'Vascular Endothelial Growth Factor A', 'Vascular development', 'Vascular Endothelial Growth Factor Receptor-1', 'Vascular Remodeling'. Only articles written in English or Chinese are included.

### Study selection

#### Inclusion and exclusion criteria

**Fig 1:**
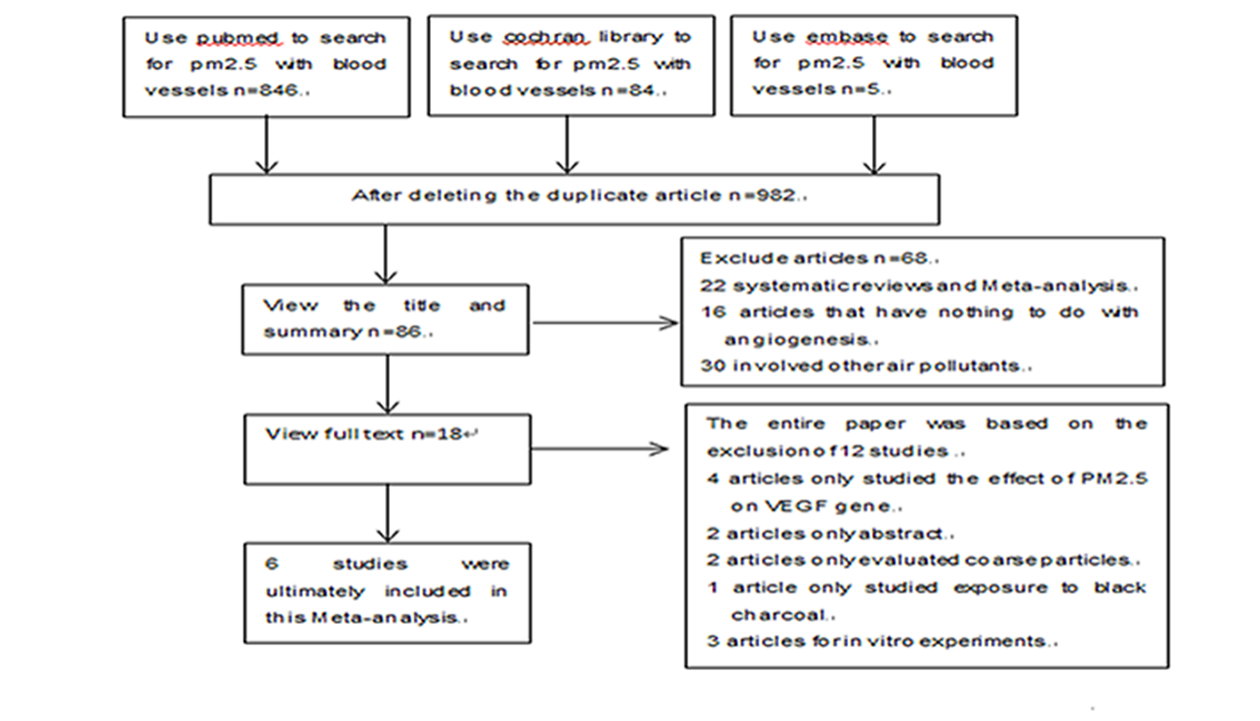
Flowchart of two methods and the study selection process.

Studies that did not contain the association between PM2.5 and angiogenesis were excluded after the preliminarily pre-screening. The rest of the studies were further assessed by the author. If the study met the following criteria, the study was included in the Meta-analysis: (a)The study included PM2.5 exposure, and the outcome measured VEGF; (b)The study provided the sample size, partial regression coefficient (β), and 95% CI of VEGF monitoring, or information that could be used to infer these results; (c)Excluding non-human studies, if more than one study was identified for the same population, only studies that included the latest population or the most information were selected. Accordingly, studies that did not meet the above criteria were excluded. The study selection process is presented in detail in Fig 1.

#### Data extraction

Information extracted from identified studies included: the author, the study site, the study time, the exposure measurement method, the data source, the number of participants, the exposure pattern, the exposure mean and range, the exposed chemical composition, the mean of VEGF, the standard deviation and confidence interval of VEGF. If the study provided a link between PM2.5 exposure and VEGF, all estimates were extracted. Some studies were based on monitoring network data and personal exposure data monitoring to assess PM2.5 exposure. In addition, estimates were extracted from a single pollutant model only with the other covariates fully adjusted, air pollutants (CO, SO2, O3, NO2) other than PM2.5 are excluded because there is considerable collinearity between the pollutants from the same source. Qualification assessment and all data extraction were carried out by the author using standard forms.

#### Meta-analysis and statistical analysis

Before Meta-analysis, we converted all the risk estimates (partial regression coefficients:β) of PM2.5 mass into common exposure units, which allowed us to summarize values for different studies. We set a common effect unit with an increase in PM2.5 mass concentration of 10 μg / m3. The correlation between particulate matter contamination and vascular endothelial growth factor was analyzed by linear mixed model or generalized linear model. Thus, based on the hypothesis of the two linear models, We combine all the values according to a uniform standard, with an increase in particulate matter per 10 μg/m^3^, an average change in VEGF and a 95% *CI*.

We then performed several Meta-analysis studies to quantitatively estimate the association of PM2.5 exposure to VEGF. A number of subgroup analyses were also performed to estimate the effect of PM2.5 exposure on VEGF with different exposure measurement methods, study design, biomarker measurement sources and countries. These subgroup analyses were designed to investigate the impact of these characteristics on PM2.5 exposure on VEGF and to further tested their effect on the heterogeneity of the reported association.

We identified two exposure measurement methods: individual level and regional level exposure assessment. Individual-level exposure assessment used personal monitor, exposure equipment testing, or time-space land using regression model prediction, etc [28–29]. These models can be highly accurate to estimated the daily PM2.5 exposure level for each subject. The regional level exposure was calculated using an air quality monitor in the area with an average PM2.5 concentration or a grid with a low resolution [24–27] and assumes that all subjects in the region are exposed to the same PM2.5 concentration. All research studies included in the study were divided into two categories: retrospective and prospective study. VEGF was measured by two methods, one for blood collection analysis for measuring VEGF and the other for collecting subjects with urine measurements of VEGF levels.

In order to explore the possible heterogeneity of the results, we assumed that the effect magnitude may vary depending on the quality of the methodology studied. The heterogeneity of the study included was assessed using the Q statistic and I^2^ statistic. Cochran's Q statistic was calculated by summing the squares of the deviations of the values from each study from the overall meta- analysis by weighting each study's contribution. P values were obtained by comparing the chi-square distribution of Q-statistic and k-1 degrees of freedom, where k was the number of included studies [34]. If *P* <0.05, then selected the random effect model, otherwise chose the fixed-effect model. The I^2^ statistics I^2^ = (Q-df)/Q x 100 describes the percentage of inter-research variability due to heterogeneity rather than contingency. The I^2^> 50% value indicates a statistically significant heterogeneity [34]. Publication bias and sensitivity analysis used Begg's and Egger's tests to apply sensitivity analysis modules to sensitivity analysis of included literature, to observe the effect of a single study on the overall effect. All statistical tests were bilateral, and *P* <0.05 was considered statistically significant. We use stata 12.0 software for analyze the data and publication offset analysis.

## Results

### Search results and study characteristics

A total of 86 studies were selected as potentially eligible publications. 68 studies were excluded (22 systematic reviews or meta-analyses, 16 articles were not related to angiogenesis, 30 articles involved other air pollutants). After an in-depth review, 12 out of the remaining 18 studies were ruled out: effect on VEGF gene (n = 3), only summary (n = 2), coarse particles (n = 2), in vitro experiments (n = 3), exposure to black carbon (n = 1). Finally, this meta-analysis included a total of 527 participants from six studies conducted in the United States, Canada and China [24–29].The details of all included studies are shown in Table 1.

**Table 1:** Characteristics of the studies included in the meta-analysis.

Author	Location	Study duration	Exposure Measurement method	Data source	No. of participants	Exposure range mean (*IQR*) / (*SD*) μg/m3	PM2.5 chemical constituents	Exposure mode	Study design	Biomarker measurement source
Pope, C. A. III et al	Utah USA	2013- 2015	Regional level	Monitoring network data	72	0–130		24h	Prospective	Blood
Pelletier, G. et al	Ottawa, Ontario, Canada	2010	Regional level	Monitoring network data	58	10.8 (3.5–27.6)	CO SO2 O3 NO2 NO	Lag 0	Prospective	Urine
Liu, L. et al	Windsor Ontario, Canada	2007	Individual level	Personal monitor	28	6.3 (7.1)		24h	Prospective	Blood
Liu, L. et al	Canada Toronto	2013	Individual level	Personal monitor	20	238.4 ±62	CO SO2 O3 NO2	1h 21h	Prospective	Blood and urine
Niu, J. et al	China Gansu	2010	Regional level	Exposure equipment testing	20	54.5±39.8	Ni, As, Se, Cu	24h	Prospective	Blood
Naveed, B. et al	NewYork, USA	2011	Regional level	Monitoring network data	271	-		24h	Retrospective	Blood

### Comprehensive estimation of the effects of the PM2.5 mass concentrations on VEGF

**Fig 2:**
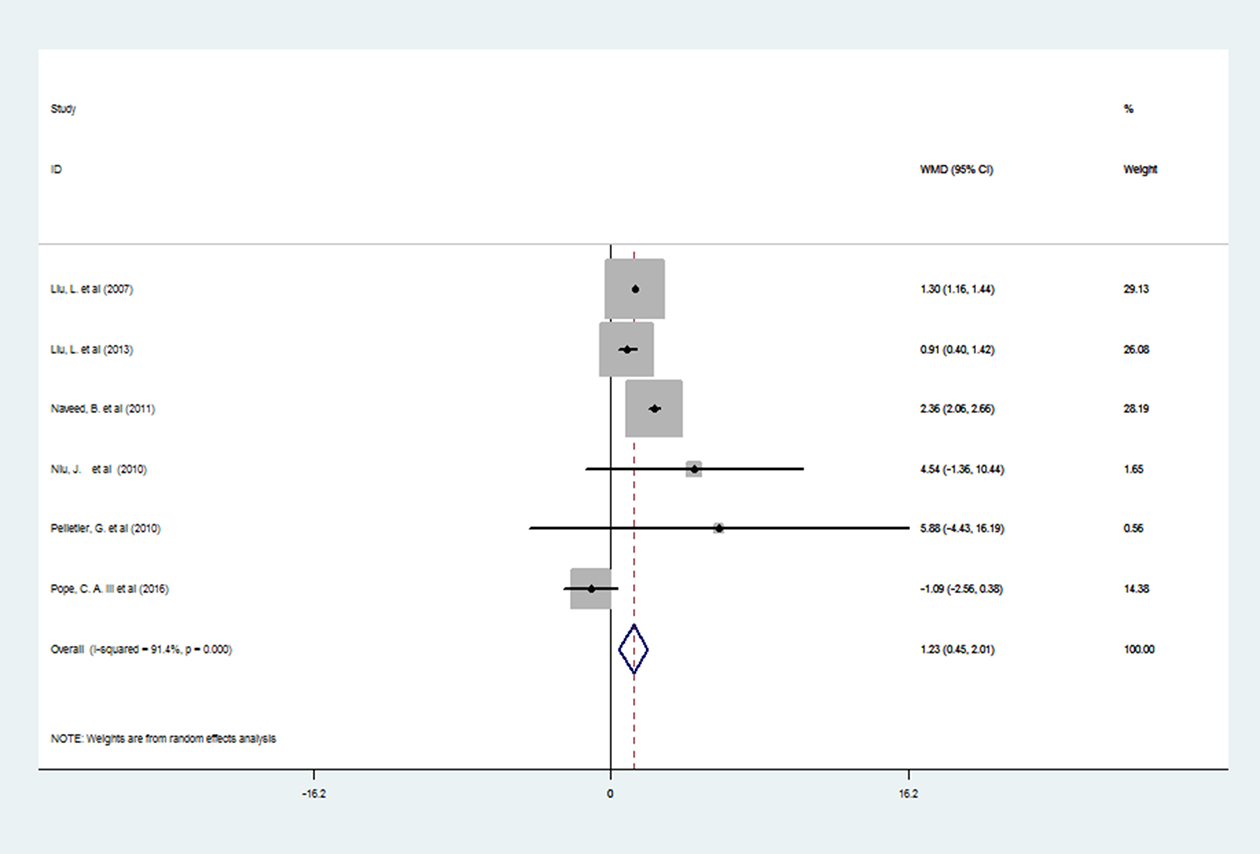
Forest plots for the association between PM2.5 exposure (per 10 μg/m3 increments) and VEGF(β,95%CI). The association between PM2.5 exposure and VEGF.β indicates changes in VEGF.

**Fig 3:**
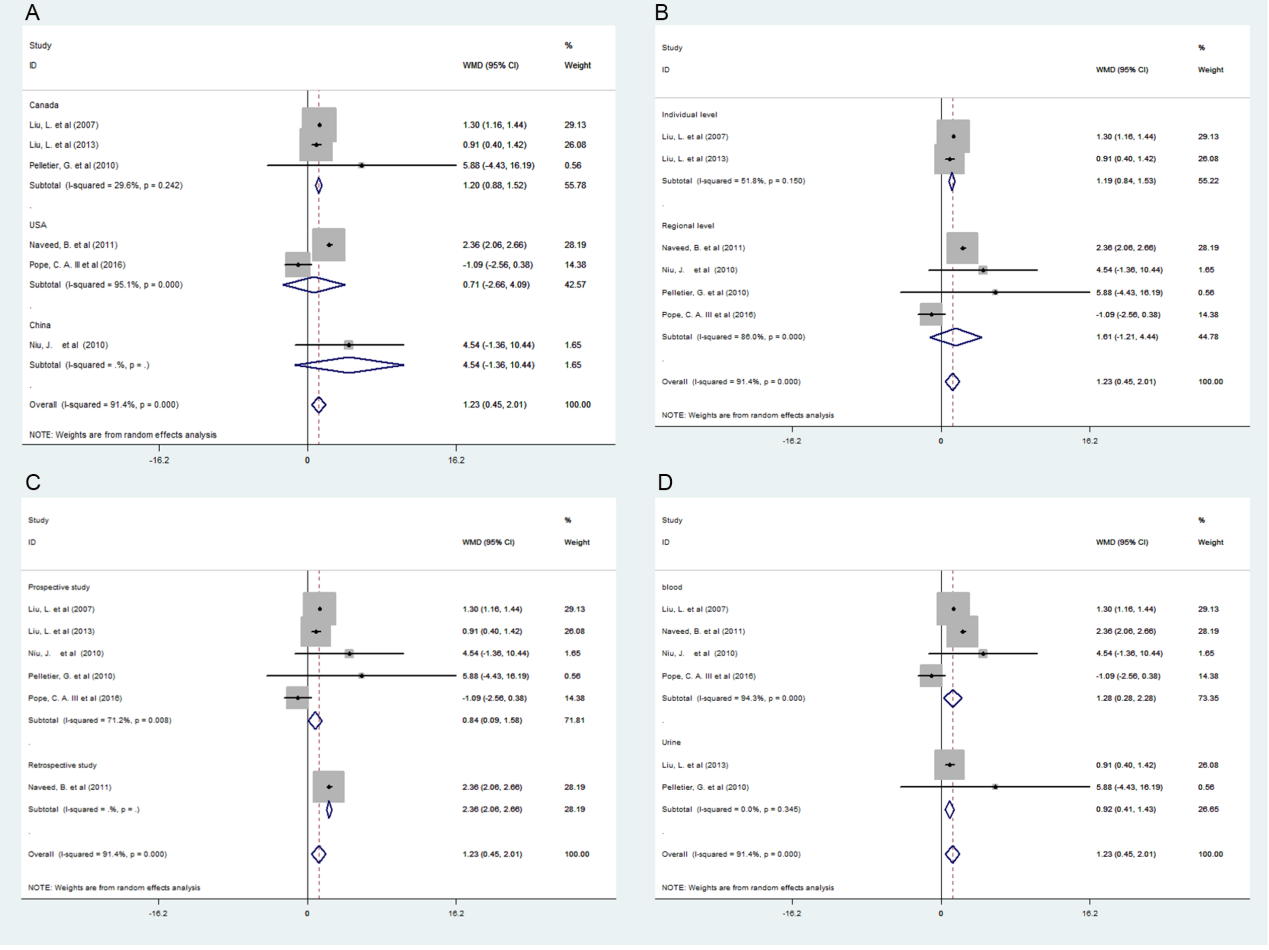
Forest plots for the association between PM2.5 exposure (per 10 μg/m3 increments) and VEGF(β,95%CI) in different subgroups. (A) The association of PM2.5 exposure and VEGF in Canada, the United States, and China. (B) The association of PM2.5 exposure and VEGF at the individual level with the regional level. (C) The association of PM2.5 exposure and VEGF in the prospective and retrospective study. (D) The association of PM2.5 exposure and VEGF in blood and urine.

It was estimated that the increase in VEGF (β = 1.23 pg/ml, 95% *CI*: 0.45, 2.01) was significantly positive associated with the PM2.5 mass concentration of 10 μg/m^3^ (Fig 2), and there was significant heterogeneity among the six studies (*P* = 0.002), as shown in Table 2. Studies from Canada showed that PM2.5 exposure statistically elevated the level of VEGF level that an increase of 1.20 pg/ml (95% *CI*: 0.88, 1.52) in VEGF was positive associated with per 10 μg/m^3^ increase in PM2.5 concentration (Fig 3A).

**Table 2:** The association between PM2.5 exposure (per 10 μg/m3 increments) and VEGF (β, 95% CI) in different subgroups.

Subgroups	No.of studies	*P* for heterogeneity test	Summary *β*(95% *CI*)	*P* for hypothesis test	*I*^*2*^(%)
All exposed	6	<0.001	1.23 [0.45, 2.01]^*^	0.002	91
Exposure assessment method					
Individual level	2	0.15	1.19 [0.84, 1.53]^*^	<0.0001	52
Regional level	4	<0.001	1.61 [-1.21, 4.44]	0.26	86
Research design					
Prospective study	5	0.008	0.84 [0.09, 1.58]^*^	0.03	71
Retrospective study	1		2.36 [2.06, 2.66]^*^	<0.0001	
Country					
Canada	3	0.24	1.20 [0.88, 1.52]^*^	<0.0001	30
USA	2	<0.001	0.71 [-2.66, 4.09]	0.68	95
China	1		4.54 [-1.36, 10.44]	0.13	
Biomarker measurements					
Urine	2	0.35	0.92 [0.41, 1.43]^*^	0.0004	0
Blood	4	<0.001	1.28 [0.28, 2.28]^*^	0.01	94

***Note*:** βindicates changes in VEGF (10 μg/m^3^,95%*CI*): *P* < 0.05.

In order to explore the source of heterogeneity among studies, a series of subgroup analyses were conducted. The results showed that PM2.5 exposure had positive impact on VEGF. In the individual levels (β = 1.19 pg/ml; 95% *CI*: 0.84, 1.53), (Fig 3B), prospective study (β = 0.84 pg/ml; 95% *CI*: 0.09, 1.58), retrospective study (β = 2.36 pg/ml; 95% *CI*: 2.06, 2.66) (Fig 3C), biomarker study in blood (β = 1.28 pg/ml; 95% *CI*: 0.28, 2.28), and urine biomarker (β = 0.92 pg/ml; 95% *CI*: 0.41, 1.43), as shown in Fig 3D. Subgroup analyses of exposure measures also found that research heterogeneity might be caused by regional exposure. On the other hand, countries were also the reasons for studying heterogeneity. In particular, three studies from Canada were included in the Meta-analysis, PM2.5 concentration increased by 10 μg/m^3^, VEGF increased by 5.88 pg/ml (95%*CI*: -4.43, 16.19), 1.30 pg/ml(95%*CI*: 1.16, 1.44) and 0.91 pg/ml(95%*CI*: 0.40, 1.42), and their combined value was 1.20 pg/ml (95%*CI*: 0.88, 1.52).

### Sensitivity analyses and publication bias analyses

**Fig 4:**
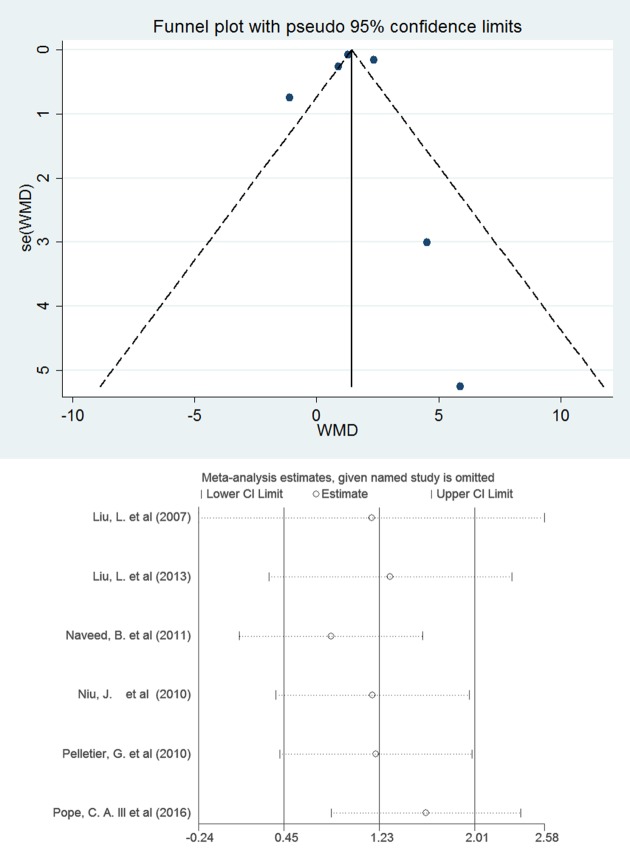
Funnel plot and sensitivity analyses: The association between PM2.5 exposure (per 10 μg/m3 increments) and VEGF(β, 95%CI).

Finally, we conducted a series of sensitivity analyses to detect the robustness of the results. We removed the largest and smallest values from a single study separately in the meta-analysis, and there was no significant change in the aggregated effect among the published data. In this study, Stata 12. 0 software was used to analyze the biases of the six articles included in the analysis. The results showed that neither Begg's test (*P* = 1) nor Egger' s test(t = 0.48,*P* = 0.681) found obvious publication bias in the included articles.(Fig 4).

## Discussion

In the present meta-analysis, we collected six eligible studies that quantitatively evaluated the relationship between PM2.5 concentration and VEGF expression, and a total of 527 subjects were included. Our results confirmed the positive association between PM2.5 exposure and VEGF level. In studies conducted in Canada, the effects of PM2.5 exposure on VEGF were statistically significant. The level of VEGF measured both in blood and urine showed that PM2.5 exposure was positive associated with VEGF elevation. We conducted a series of subgroup analyses, and the results showed that the measurement method of the exposure and the country the study was conducted likely contributed to the heterogeneity. The number of related studies was limited. Thus, it is necessary to make further meta-analysis to explore the origin of heterogeneity, and more original studies will be included in the future.

Exposure to particulate matter can lead to neovascularization [35]. Neovascularization promotes malignant tumors and cardiovascular disease and involves in the formation and progress of the other diseases. VEGF is a major factor in angiogenesis that promotes angiogenesis and induces vascular remodeling [36–37].Therefore, a better understanding of the correlation between PM2.5 exposure and VEGF levels might shed some light on the role PM2.5 plays in inducing cardiovascular revascularization and tumor angiogenesis.

We collected articles on the impact of PM10 or black carbon on VEGF [38–39], while the number of the studies was too few to allow meta-analysis to address the relevance. In addition, we also attempted to collect and incorporate four articles studied cells exposed to PM2.5 in vitro [40–43]. However, these studies were conducted in a higher concentration of PM2.5 (0.1 to 1 μg/ml), besides, the sample size and effect values were very small after converting to the same unit as our study. On the other hand, only one cell line was used in the experiment, and the basic can be assumed that the object is completely homogeneous, which does not accord with the diversity among human individuals. So it is not recommended to combine data from in vivo and in vitro experiments in analysis.

Our Meta-analysis showed that PM2.5 exposure had significant effect on VEGF in the blood and urine. It has been suggested that in the PM2.5 exposure experiment, the level of VEGF can be indirectly measured by collecting VEGF biomarkers in the urine, providing new ideas for the detection of VEGF as a biomarker for cardiovascular disease induced by PM2.5.

We performed a series of subgroup analyses, and the results showed it was the regional level exposure that caused the correlation heterogeneity between PM2.5 exposure and VEGF. It has been suggested that the PM2.5 exposure assessment at the regional level may lead to misrepresentation of the exposure, as it does not take into consideration the spatial misalignment between the individual’s residence and the monitoring point, and it ignores the fact that the individuals have different patterns of activity (indoor and outdoor activities). In contrast, Individual-level methods include risk, meteorology, road geometry, vehicle emissions, air quality monitoring data, and land use information. The use of personal monitors to assess individual exposure to PM2.5 levels can significantly reduce the exposure assessment bias.

The heterogeneity test revealed that the association heterogeneity between PM2.5 exposure and VEGF was most likely due to different countries where the studies were conducted. The source, the toxicity and the impact of PM2.5 on health may vary as geographical area. Therefore, it is reasonable to perform subgroup meta-analysis to test the variation of PM2.5 estimates among regions. The three countries included in the present study were Canada, the United States, and China, and the estimates for VEGF in PM2.5 in the three countries were different: β = 1.20 pg/ml; 95%*CI*: 0.88, 1.52), (β = 0.71 pg/ml; 95%*CI*: -2.66, 4.09) and (β = 4.54 pg/ml; 95%*CI*: -1.36, 10.44). This difference may be related to changes in population, environment, or PM2.5 composition in the three regions. For example, PM2.5 composition in China contains more Ni, Cu, other organic and inorganic components, which might partly lead to the larger value. Since most of the articles did not mention the main components of PM2.5 exposure in the region, our research has also been limited. In addition, the amount of research may be another important factor. During the process of research selection, only four studies conducted in China investigated the association between PM2.5 exposure and VEGF level. Among which, three were carried out with in vitro cell lines and mRNA level of VEGF was measured [41–43]. Thus, only one study from China detected the VEGF level in the blood or urine was included in the analysis. China has been suffering from severe PM2.5 contamination over the past few decades, but the relationship between PM2.5 and VEGF was remained elusive, as the studies, especially epidemiological studies, addressing this issue were very few. The present meta-analysis might be able to provide some specific information and shed a light on the public health issue. We think it’s necessary to do further study to elucidate the possible underlying mechanisms of cardiovascular diseases and cancer caused by PM2.5 exposure and the potential use of VEGF an easy-to-detect biomarker of cardiovascular diseases caused by PM2.5 exposure. Hopefully, the work we did could offer information for policymakers and public health practitioners to predict the health effects of air pollution.

This is the first analysis estimated the correlation between airborne fine particulate matter and VEGF. Secondly, the Meta-analysis focused on studies in both developed and developing countries: the United States, Canada and China The limitation of this meta-analysis was the high or moderate heterogeneity in most subgroup meta-analyses, although less heterogeneity was found in some subgroups. These results suggested that the heterogeneity could be affected by other factors, such as economic conditions, and a limited number of related studies. Thus, more Meta-analysis is necessary to explore the origin of the heterogeneity, and more original studies will be conducted in the future.

## Conclusion

In conclusion, this meta-analysis revealed a positive correlation between PM2.5 exposure and VEGF level. Exposure assessment methods and study countries were the major sources of heterogeneity among studies. These results extended our understanding of the adverse effects of exposure to PM2.5 that caused angiogenesis and vascular remodeling by increasing the levels of VEGF. More research are needed in the future to assess the adverse effects of PM 2.5 exposures on VEGF in countries other than Canada, particularly in developing countries.

## Supporting information

S1 File PRISMA 2009 checklist. (DOCX)
