# Supplementary material for: Exposure to PM2.5 via vascular endothelial growth factor relationship: Meta-analysis
Source: PLoS One. 2018 Jun 18;13(6):e0198813. doi: 10.1371/journal.pone.0198813 (PMC6005507; doi:10.1371/journal.pone.0198813)
Supplement: S1 File — (DOCX) [file pone.0198813.s001.docx]

**Exposure to particulate matter (PM2.5) via vascular endothelial growth factor (VEGF) relationship: Meta-analysis**

Yi Sun^1^,Yao Wang^1^, Shu Yuan^2^,Jialing Wen^3^,Weiyu Li^4^,Liu Yang^1^,Xiaoyan Huang^1^,Yanmei Mo^1^,Yingqi Zhao^1^, Yuanming Lu^1*^

1. Department of Toxicology, Guilin Medical University School of Public Health, Guilin, 541004, China.

2. The library and information center, China pharmaceutical university, Nanjing, 210000, China.

3. Guangdong Provincial Key Laboratory of Colorectal and Pelvic Floor Diseases. The Sixth Affiliated Hospital, Sun Yat-sen University, Guangzhou, 51000, China.

4. 181st Hospital of People's Liberation Army of China, Guilin,541004, China.

Yi Sun, Yao Wang and Shu Yuan contributed equally to this work.

*Corresponding author: Prof. Yuanming Lu, Department of Toxicology, Guilin Medical University School of Public Health, Guilin, 541004, China. Phone: +86-0773-5895812; Fax: +86-0773-5895812. Email: [bobojpn2002@163.com](mailto:bobojpn2002@163.com).

**ABSTRACT**

We performed Meta-analysis to estimate the association of PM2.5 exposure and VEGF, and to explore the association of these exposed source heterogeneity. We searched all the studies published in the Cochrane Library, PUBMED, Embase, China National Knowledge Infrastructure China National Knowledge Infrastructure, and WanFang Electronic Database before June 2017. Finally six studies were identified. It confirmed that increase in VEGF level was statistically significant for 10 μg/m^3^ increasing of PM2.5 mass concentration (β= 6.25 pg/ml; 95% *CI*: 3.82, 9.22). The results of the PM2.5 exposure from Canada and China were estimated, and it was found that the increase of PM2.5 concentration was 10μg / m3, VEGF increased 0.60 pg/ml (95% *CI*: 0.47, 0.74) and 4.79 pg/ml (95%*CI*: 2.00, 7.58). Other subgroup analyses indicated that the different effects of PM2.5 exposure on VEGF in different exposure assessment methods, study designs, and study settings. It was conclude that a clear association between PM2.5 exposure and VEGF levels. Exposure assessment methods and study countries were the important sources of heterogeneity among studies.

**Key Word:** PM2.5; VEGF; Angiogenesis; Meta-analysis

**INTRODUCTION**

Environmental particulate matter (PM2.5) refers to particulates with atmospheric aerodynamic diameters less than 2.5 microns, also known as inhalable lung particles, which have an important impact on human health and atmospheric environmental quality. PM2.5 pollution in China has attracted attention from all of the world. According to Chinese environmental monitoring report, there was three large-scale regional haze pollution breaking up, including heavy pollution for 155 days and serious pollution for 31 days in 161 cities only from October 1 to October 24 in 2016, PM2.5 in multiple cities was over 1000^[1]^. The epidemiological results have confirmed that long-term exposure to PM2.5 has been linked to the mortality rising in the population ^[1]^ and promotes the incidence of lung cancer increasing ^[2-4]^. In addition, PM2.5 exposure is also a risk factor for cardiovascular disease ^[5]^, and PM2.5 caused cardiovascular disease via vascular endothelial dysfunction,^[6]^ mediator of diastolic blood flow, accelerating the progression of atherosclerotic plaques. ^[7]^

More and more clinical trials have found that exposure to environmental fine particles increases neovascularization ^[8]^, and it is known that angiogenesis is essential for the occurrence and development of cardiovascular disease and tumors ^[9-11]^. Angiogenesis and vascular remodeling lead to a variety of cardiovascular diseases such as myocardial ischemia, peripheral arterial disease, atherosclerosis and aortic aneurysm ^[12-13]^. Studies have shown that exposure to fine particulate air pollution caused cardiovascular remodeling, and finally made cardiac phenotype which was similar to initial the heart failure. ^[14]^Angiogenesis are thought to be a critical process of tumor growth, and metastasis and pathologic angiogenesis are a sign of cancer and various ischemic and inflammatory diseases ^[15-18]^.

VEGF is the most potent growth factor in blood vessel so far, which induces endothelial cells proliferation and capillary formation and is one of the major components taking part in vascular remodeling ^[19-20]^. The level of VEGF rising may be a result to acute systemic reactions in endothelial injury after exposure to PM and endotoxin, and then VEGF strongly increases the proliferation of endocardial cells, significantly induced myocardial and endocardial growth rate damage ^[21]^. Abnormal elevation of local VEGF expression plays an important role in the regulation of angiogenesis in the pathogenesis of heart disease and the angiogenesis of the tumor. ^[22-23]^ However, the correlation between PM2.5 exposure and VEGF is not consistent ^[24-29]^. These inconsistent and controversial results suggest that the need for quantitative synthesis and interpretation of the results of various studies to determine their relevance, moreover to explore the role and the mechanism of VEGF induced cardiovascular disease after exposure to PM2.5 and whether VEGF could be a biomarker of cardiovascular diseases caused by PM2.5.

Meta-analysis is the most common statistical technique used for quantitative and comprehensive analysis of results from two or more individual studies ^[30]^. To more accurately estimate the association of PM2.5 exposure with VEGF, we conducted a Meta-analysis of the relationship between PM2.5 and VEGF in all published studies. In this analysis, we systematically collected previously published studies to estimate the effect of PM2.5 exposure on VEGF, and then used the Mate-analysis model to quantitatively assess the effect of exposure to PM2.5 on VEGF. We further explored the effects of exposure measurement methods, study design, biomarker measurement sources, and count on the meta-estimation of PM2.5.

**MATERIALS AND METHODS**

We searched all the studies published in the Cochrane Library, PUBMED, Embase, China National Knowledge Infrastructure, and Wanfang Electronic Database before June 2017. Our search strategy used the following PM2.5 keywords: 'air pollution', 'particulate matter', 'ambient particulate matter', 'PM', 'PM2.5', 'PM10', 'airborne particulate matter', 'particulate air pollutants', 'black carbon', 'BC（black carbon）', as well as neovascularization: 'neovascularization', 'Angiogenesis, Pathologic', 'Vascular Endothelial Growth Factor A', 'Vascular development', 'Vascular Endothelial Growth Factor Receptor-1', 'Vascular Remodeling'. We also manually searched for additional publications for each of the major studies. Further publications were also reviewed by reviewing the article. Only English or Chinese publications are included.

***Study selection***

***Inclusion and exclusion criteria*** We initially screened all the articles and profiles of the study, and if the study did not involve the association between PM2.5 and angiogenesis, the study was excluded. The rest of the studies were marked as qualifying and further assessed by the author. If the study met the following criteria, the study was included in the Meta-analysis: (a)The study included PM2.5 exposure, and the outcome measured VEGF; (b)The study provided the sample size, partial regression coefficient (β), and 95% *CI* of VEGF monitoring, or information that could be used to infer these results; (c)Excluding non-human studies, if more than one study was identified for the same population, only studies that included the latest population or the most information were selected. Accordingly, studies that did not meet the above criteria were excluded. The study selection process is presented in detail in Fig 1.

***Data extraction*** The following information was extracted from each study: the author, the study site, the study time, the exposure measurement method, the data source, the number of participants, the exposure pattern, the exposure mean and range, the exposed chemical composition, the mean of VEGF, the standard deviation and confidence interval of VEGF. If the study provided a link between PM2.5 exposure and VEGF, all estimates were extracted. Some studies were based on monitoring network data and personal exposure data monitoring to assess PM2.5 exposure. In addition, estimates were extracted from a single pollutant model only with the other covariates fully adjusted, air pollutants (CO, SO2, O3, NO2) other than PM2.5 are excluded because there is considerable collinearity between the pollutants from the same source. Qualification assessment and all data extraction were carried out by the author using standard forms.

***Meta-analysis and statistical analysis***

Before Meta-analysis, we converted all the risk estimates (partial regression coefficients:β) of PM2.5 mass into common exposure units, which allowed us to summarize values for different studies. We set a common effect unit with an increase in PM2.5 mass concentration of 10 μg / m3. The correlation between particulate matter contamination and vascular endothelial growth factor was analyzed by linear mixed model or generalized linear model. Thus, based on the hypothesis of the two linear models, We combine all the values according to a uniform standard, with an increase in particulate matter per 10 μg/m^3^, an average change in VEGF and a 95% *CI*.

**Fig 1. Flowchart of two methods and the study selection process.**

We then performed several Meta-analysis studies to quantitatively estimate the association of PM2.5 exposure to VEGF. A number of subgroup analyses were also performed to estimate the effect of PM2.5 exposure on VEGF with different exposure measurement methods, study design, biomarker measurement sources and subgroups of countries. These subgroup analyses were designed to investigate the impact of these characteristics on PM2.5 exposure on VEGF and to further tested their effect on the heterogeneity of the reported association.

We identified two exposure measurement methods: individual level and regional level exposure assessment. Individual-level exposure assessment used personal monitor, exposure equipment testing, or time-space land using regression model prediction, etc. ^[28-29]^ These models can be highly accurate to estimated the daily PM2.5 exposure level for each subject. The regional level exposure was calculated using an air quality monitor in the area with an average PM2.5 concentration or a grid with a low resolution ^[24-27]^ and assumes that all subjects in the region are exposed to the same PM2.5 concentration. All research studies included in the study were divided into two categories: retrospective and prospective study. VEGF was measured by two methods, one for blood collection analysis for measuring VEGF and the other for collecting subjects with urine measurements of VEGF levels.

In order to explore the possible heterogeneity of the results, we assumed that the effect magnitude may vary depending on the quality of the methodology studied. The heterogeneity of the study included was assessed using the Q statistic and I^2^ statistic. Cochran's Q statistic was calculated by summing the squares of the deviations of the values from each study from the overall meta- analysis by weighting each study's contribution. P values were obtained by comparing the chi-square distribution of Q-statistic and k-1 degrees of freedom, where k was the number of included studies ^[31]^. If *P* <0.05, then selected the random effect model, otherwise chose the fixed-effect model. The I^2^ statistics [I^2^=(Q-df)/Q x 100] describes the percentage of inter-research variability due to heterogeneity rather than contingency. The I^2^> 50% value indicates a statistically significant heterogeneity. ^[31]^ Publication bias and sensitivity analysis used Beg's and Egger's tests to apply sensitivity analysis modules to sensitivity analysis of included literature, to observe the effect of a single study on the overall effect. All statistical tests were bilateral, and *P* <0.05 was considered statistically significant. We used Review Manager 5.3 software to analyze the data and stata 12.0 software for publication offset analysis.

**RESULTS**

***Search results and study characteristics***

A total of 86 studies was selected as potentially eligible publications. After excluded 68 studies (22 were systematic reviews or Meta-analyses, 16 articles related to angiogenesis, 30 articles involved other air pollutants), 18 studies were identified for the more detailed assessment. Further ruled out 12 studies: effect on VEGF gene (n = 3), only summary (n = 2), coarse particles (n = 2), in vitro experiments (n = 3), study exposure to black carbon (n=1). Finally, this Meta-analysis included six studies and 1116 participants ^[24-29]^. Research countries include the United States, Canada, China. The details of all included studies are shown in Table 1.

**Table 1．Characteristics of the studies included in the Meta-analysis**

| Author | Location | Study  duration | Exposure Measurement method | Data source | No. of participants | Exposure range mean （*IQR*）/（*SD*） μg/m3 | PM2.5 chemical constituents | Exposure mode | Study design | Biomarker measurement source |
| --- | --- | --- | --- | --- | --- | --- | --- | --- | --- | --- |
| Pope, C. A. III et al | Utah USA | 2013-  2015 | Regional level | Monitoring network data | 355 | 10 |  | 24h | Prospective | Blood |
| Pelletier, G. et al | Ottawa, Ontario, Canada | 2010 | Regional level | Monitoring network data | 58 | 10.8 (10.845） | CO SO2 O3 NO2 NO | Lag 0 | Prospective | Urine |
| Liu, L. et al | Windsor Ontario, Canada | 2007 | Individual level | Personal monitor | 28 | 6.3 (7.1 ） |  | 24h | Prospective | Blood |
| Liu, L. et al | Canada Toronto | 2013 | Individual level | Personal monitor | 20 | 23.84 ±6.2 | CO SO2 O3 NO2 | 1h 21h | Prospective | Blood and urine |
| [Niu, J. et al](https://www.ncbi.nlm.nih.gov/pubmed/?term=Niu%20J%5BAuthor%5D&cauthor=true&cauthor_uid=24386277) | China Gansu | 2010 | Regional level | Exposure equipment testing | 60 | 47.4±38.9 | Ni, As, Se ,Cu | 24h | Prospective | Blood |
| Naveed, B. et al | NewYork, USA | 2011 | Regional level | Monitoring network data | 271 | - |  | 24h | Retrospective | Blood |

***Comprehensive estimation of the effect of the PM2.5 mass concentrations on VEGF***

It was estimated that the increase in VEGF (β = 6.25 pg/ml, 95% *CI*: 3.82, 9.22) was associated with a PM2.5 mass concentration in 10 μg/m^3^ and was statistically significant(Fig 2-A),and there was an important study heterogeneity between the six studies (*P* < 0.001), as shown in Table 2. Studies from Canada and China PM2.5 exposure to VEGF had a statistically significant, with an increase of 0.60 pg/ml (95% *CI*: 0.47, 0.74) and 4.79 pg/ml (95％*CI*: 2.00, 7.58) for every 10 μg/m^3^ increase in PM2.5 concentration (Fig 2-B).

**Table 2. The association between PM2.5 exposure (per 10 μg/m^3^ increments) and VEGF (**β**, 95% *CI*) in different subgroups**

| **Subgroups** | **No.of studies** | ***P* for heterogeneity test** | **Summary *β*(95% *CI*)** | ***P* for hypothesis test** | ***I^2^*(%)** |
| --- | --- | --- | --- | --- | --- |
| All exposed | 6 | <0.001 | 6.25 [3.82, 9.22]^*^ | <0.0001 | 98 |
| Exposure assessment method |  |  |  |  |  |
| Individual level | 2 | 0.99 | 0.91 [0.40, 1.42]^*^ | 0.0005 | 0 |
| Regional level | 4 | <0.001 | 8.65 [0.40, 16.90]^*^ | 0.04 | 99 |
| Research design |  |  |  |  |  |
| Prospective study | 5 | <0.001 | 2.22 [0.98, 3.46]^*^ | 0.0004 | 89 |
| Retrospective study | 1 |  | 23.60 [20.62, 26.58]^*^ | <0.0001 |  |
| Country |  |  |  |  |  |
| Canada | 3 | 0.47 | 0.60 [0.47, 0.74]^*^ | <0.0001 | 0 |
| USA | 2 | <0.001 | 14.75 [-2.54, 32.03] | 0.09 | 99 |
| China | 1 |  | 4.79 [2.00, 7.58]^*^ | 0.0008 |  |
| Biomarker measurements |  |  |  |  |  |
| Urine | 2 | 0.22 | 0.65 [0.38, 0.92]^*^ | <0.001 | 33 |
| Blood | 5 | <0.001 | 8.45 [0.08, 16.97]^*^ | 0.05 | 99 |

***Note:*** βindicates changes in VEGF（10 μg/m^3^,95％*CI*）: *P* < 0.05.

**Fig 2. Forest plots for the association between PM2.5 exposure (per 10 μg/m3 increments) and VEGF(β,95%CI).**

(A)The association between PM2.5 exposure and VEGF.βindicates changes in VEGF. (B) The association of PM2.5 exposure and VEGF in Canada, the United States, and China. (C) The association of PM2.5 exposure and VEGF at the individual level with the regional level. (D) The association of PM2.5 exposure and VEGF in the prospective and retrospective study. (E) The association of PM2.5 exposure and VEGF in blood and urine.

In order to explore the source of heterogeneity between studies, a series of subgroup analyses were conducted. The results showed that PM2.5 exposure has a significant impact on the study of VEGF in studies with different exposure measurement methods, study design, biomarker measurement sources and national studies. In the evaluation of PM2.5 individual levels (β = 0.91 pg/ml ; 95% *CI*: 0.40, 1.42), PM2.5 regional level (β = 8.65 pg/ml; 95% *CI*: 0.40, 16.90)(Fig 2-C), prospective study (β= 2.22 pg/ml; 95% *CI*: 0.98, 3.46), retrospective study (β = 23.6 pg/ml ; 95% *CI*: 20.62,26.58) (Fig 2-D), biomarker study in blood (β = 8.45 pg/ml; 95% *CI*: 0.08, 16.97), urine biomarker (β = 0.65 pg/ml ; 95% *CI*: 0.38, 0.92) ,as shown in Fig 2-E. We found that PM2.5 exposure has a significant impact. Subgroup analyses of exposure measures also found that research heterogeneity may be caused by regional exposure .On the other hand, countries were also the reasons for studying heterogeneity. In particular, three studies from Canada were included in the Meta-analysis, PM2.5 concentration increased by 10 μg/m^3^, VEGF increased by 0.58 pg/ml (95％*CI*：0.44, 0.72), 1.30 pg/ml(95％*CI*: -91.42, 94.02) and 0.91 pg/ml(95％*CI*: 0.40, 1.42), and their combined value was 0.60 pg/ml (95％*CI*:0.47, 0.74).

***Sensitivity analyses and publication bias analyses***

Finally, we conducted a series of sensitivity analyses to detect the robustness of the results. We removed the largest and smallest values from a single study separately in the Meta-analysis, and there was no significant change in the aggregated effect among the published data. In this study, Stata 12. 0 software was used to detect the biases of the six articles included in the analysis. The results showed that neither Begg's test (*P* = 0.452) nor Egger' s test(t= 1.89,*P* = 0.132) found obvious publication bias in the included articles.(Fig 3).

**Fig 3. Funnel plot of comparison: The association between PM2.5 exposure (per 10 μg/m^3^ increments) and VEGF(**β**, 95%*CI*).**

**DISCUSSION**

In this Meta-analysis, we collected six eligible studies, including 1116 subjects. And it was quantitatively evaluated the relationship between PM2.5 concentration and VEGF expression. Our results suggest that PM2.5 exposure is associated with VEGF. The effect of PM2.5 exposure on VEGF from Canada and China studies were statistically significant, however, only one study in developing countries was available. The level of VEGF biomarkers measured both in blood and urine showed that PM2.5 was associated with VEGF. We conducted a series of subgroup analyses, and the results showed that the exposure measurement method and the study country likely were the cause of heterogeneity between the PM2.5 exposure and VEGF. The number of related studies was limited. Thus, it is necessary to make further Meta-analysis to explore the origin of heterogeneity, and more original studies will be included in the future.

Exposure to particulate matter can lead to neovascularization ^[32]^. Neovascularization promotes malignant tumors and cardiovascular disease and other important diseases of the formation and progress. VEGF is a major factor in angiogenesis that can increase angiogenesis and induce vascular remodeling. ^[33-34]^ Therefore, the study of the correlation between PM2.5 exposure and VEGF plays an important role in PM2.5 induced cardiovascular revascularization and tumor angiogenesis.

We collected PM10, black carbon and VEGF related studies ^[35-36]^, however, the number of studies was too small to allow Meta-analysis to discuss its relevance. In addition, because of small number of studies included, we also attempted to collect and incorporate four articles in vitro cell experiments exposed to PM2.5 ^[37-40]^. However, these studies were conducted in a higher concentration of PM2.5 (0.1 to 1 μg/ml), besides, the sample size and effect values were very small after conversion to the same unit with our study. On the other hand, only one cell line was used in the experiment, and the basic can be assumed that the object is completely homogeneous, which does not accord with the differences among human individuals. So it is not recommended that experiments in vivo and in vitro combined analysis.

Our Meta-analysis showed that the effect of PM2.5 exposure on VEGF in the blood and urine was statistically significant. It has been suggested that in the PM2.5 exposure experiment, the level of VEGF can be indirectly measured by collecting VEGF biomarkers in urine. Provide new ideas for the detection of VEGF as a biomarker for cardiovascular disease induced by PM2.5.

We performed a series of subgroup analyses, and the results showed it was the regional level exposure which caused the correlation heterogeneity between PM2.5 exposure and VEGF .It has been suggested that the PM2.5 exposure assessment at the regional level may lead to an erroneous representation of the exposure, because these methods cannot explain the spatial misalignment between the individual residence and the monitoring point, and the individual has different patterns of activity (indoor and outdoor activities). In contrast, Individual-level assessment methods exist variables which affect people exposed to PM2.5, including risk, meteorology, road geometry, vehicle emissions, air quality monitoring data, and land use information. The use of personal monitors to assess individual exposure to PM2.5 levels can significantly reduce the exposure assessment bias.

The heterogeneity test revealed that the association heterogeneity between PM2.5 exposure and VEGF was most likely due to the study country. If the source of PM2.5 was different, the toxicity and health effects of PM2.5 may vary as geographical area different. Therefore, it is reasonable to perform subgroup Meta-analysis to test the variation of PM2.5 estimates among regions. In this study, we divided all the included studies into three groups (Canada, the United States, and China). For the VEGF analysis, we observed that the estimates for VEGF in PM2.5 in three countries were different (β = 0.60 pg/ml ; 95%*CI*: 0.47, 0.74), (β = 14.75 pg/ml ; 95%*CI*:-2.54, 32.03) and (β = 4.79 pg/ml; 95%*CI*: 2.00, 7.58). This difference may be related to changes in population, environment, or PM2.5 composition in three regions. For the United States and China with a larger value, it may because PM2.5 composition in the two countries, which contains more Ni, Cu, other organic and inorganic components. Since most of the articles did not mention the main components of PM2.5 exposure in the region, our research has also been limited. In addition, the amount of research may be another important factor. We accepted only one study from China. When we collected the literature, we found that few Chinese PM2.5 exposure study detecting VEGF in intravascular or urine. Finally, we searched four articles, three of which were the effects of PM2.5 on VEGF in vitro, and observed that PM2.5 exposure was up-regulated at the gene level,^[38-40]^ thus the three articles were exclude . China has been suffering from severe PM2.5 contamination, but the study on the relationship in between PM2.5 and VEGF, especially epidemiological studies, is much less while our Meta-analysis happened to provide some specific information. We think it necessary to do the further study in this direction. So these data would not only explain the mechanism of cardiovascular and cancer caused by PM2.5 but also makes VEGF become an easy-to-detect biomarker. Moreover, important information will be offered for policymakers and public health practitioners, even help to predict the health effects of air pollution.

There are some advantages in this study. Firstly, it is the first time that the correlation between airborne fine particulate matter and VEGF correlation is analyzed. Secondly, the Meta-analysis focuses on three different countries (the United States, Canada, China), including developing and developed countries.

The limitation of this Meta-analysis is that we found that there is a high or moderate heterogeneity in most subgroup meta-analyses, although less heterogeneity is found in some subgroups. These results suggest that the heterogeneity could be affected by other factors, such as economic conditions, and a limited number of related studies. Thus, more Meta-analysis is necessary to explore the origin of heterogeneity, and more original studies will be conducted in the future.

**CONCLUSION**

In conclusion, this Meta-analysis revealed a positive correlation between PM2.5 exposure and VEGF level. Exposure assessment methods and study countries are the important sources of heterogeneity between studies. These results extend our understanding of the adverse effects of exposure to PM2.5, which increases the levels of VEGF and causes angiogenesis and vascular remodeling. More research are needed in the future to assess the adverse effects of PM 2.5 exposures on VEGF in countries other than Canada, particularly in developing countries.

**ACKNOWLWDGEMENTS**

This work was supported financially by grants from the National Natural Science Foundation of China (81460446) and Guangxi Natural Science Foundation(2015GXNSFDA139021).

**REFERENCES**

1. Krewski D, Jerrett M, Burnett RT, Ma R, Hughes E, Shi Y et al. Extended follow-up and spatial analysis of the American Cancer Society study linking particulate air pollution and mortality. Res Rep Health Eff Inst 2009; (140), 5-114;
2. Raaschou-Nielsen O, Andersen ZJ, Beelen R, Samoli E, Stafoggia M, Weinmayr G et al. Air pollution and lung cancer incidence in 17 European cohorts: prospective analyses from the European Study of Cohorts for Air Pollution Effects (ESCAPE). Lancet Oncol, 2013; 14(9), 813-822.
3. Turner MC, Krewski D, Pope CA 3rd, Chen Y, Gapstur SM, Thun MJ. Long-term ambient fine particulate matter air pollution and lung cancer in a large cohort of never-smokers. Am J Respir Crit Care Med, 2011; 184(12), 1374-1381.
4. Pope CA 3rd, Burnett RT, Thun MJ, Calle EE, Krewski D, Ito K et al. Lung cancer, cardiopulmonary mortality, and long-term exposure to fine particulate air pollution. Jama, 2002; 287(9),
5. Shanley RP, Hayes RB, Cromar KR, Ito K, Gordon T, Ahn J. Particulate Air Pollution and Clinical Cardiovascular Disease Risk Factors. Epidemiology, 2016; 27(2), 291-298.
6. Krishnan RM, Adar SD, Szpiro AA, Jorgensen NW, Van Hee VC, Barr RG et al. Vascular responses to long- and short-term exposure to fine particulate matter: MESA Air (Multi-Ethnic Study of Atherosclerosis and Air Pollution). J Am Coll Cardiol, 2012; 60(21), 2158-2166.
7. Allen RW, Criqui MH, Diez Roux AV, Allison M, Shea S, Detrano R et al. Fine particulate matter air pollution proximity to traffic and aortic atherosclerosis. Epidemiology, 2009; 20(2), 254-264.
8. Mani V, Wong SK, Sawit ST, Calcagno C, MacEda C, Ramachandran S et al. Relationship between particulate matter exposure and atherogenic profile in "ground Zero" workers as shown by dynamic contrast enhanced MR imaging. International Journal of Cardiovascular Imaging, 2013; 29(4), 827-833.
9. Folkman J. Angiogenesis in cancer, vascular, rheumatoid and other disease. Nat Med, 1995; 1(1), 27-31.
10. Liotta LA, Steeg PS, Stetler-Stevenson WG. Cancer metastasis and angiogenesis: an imbalance of positive and negative regulation. Cell, 1991; 64(2), 327-336.
11. Vijaynagar B, Bown MJ, Sayers RD, Choke E. Potential role for anti-angiogenic therapy in abdominal aortic aneurysms. Eur J Clin Invest, 2013; 43(7), 758-765.
12. Golestani R, Jung JJ, Sadeghi MM. Molecular Imaging of Angiogenesis and Vascular Remodeling in Cardiovascular Pathology. J Clin Med, 2016; 5(6).
13. Sluimer JC, Daemen MJ. Novel concepts in atherogenesis: angiogenesis and hypoxia in atherosclerosis. J Pathol, 2009; 218(1), 7-29.
14. Wold LE, Ying Z, Hutchinson KR, Velten M, Gorr MW, Velten C et al. Cardiovascular remodeling in response to long-term exposure to fine particulate matter air pollution. Circ Heart Fail, 2012; 5(4), 452-461.
15. Hanahan D, Weinberg RA. The hallmarks of cancer. Cell, 2000; 100(1), 57-70.
16. Carmeliet P, Jain RK. Angiogenesis in cancer and other diseases. Nature, 407(6801), 2000; 249-257.
17. Folkman J. What is the evidence that tumors are angiogenesis dependent? J Natl Cancer Inst, 1990; 82(1), 4-6.
18. Goel S, Duda DG, Xu L, Munn LL, Boucher Y, Fukumura D et al. Normalization of the vasculature for treatment of cancer and other diseases. Physiol Rev, 2011; 91(3), 1071-1121.
19. Chetta A, Zanini A, Foresi A, D'Ippolito R, Tipa A, Castagnaro A et al. Vascular endothelial growth factor up-regulation and bronchial wall remodelling in asthma. Clin Exp Allergy, 2005; 35(11), 1437-1442.
20. Zhou H, Binmadi NO, Yang YH, Proia P, Basile JR. Semaphorin 4D cooperates with VEGF to promote angiogenesis and tumor progression. Angiogenesis, 2012; 15(3), 391-407.
21. Feucht M, Christ B, Wilting J. VEGF induces cardiovascular malformation and embryonic lethality. Am J Pathol, 1997; 151(5), 1407-1416.
22. Haigh JJ. Role of VEGF in organogenesis. Organogenesis, 2008; 4(4), 247-256.
23. Ferrara N, Gerber HP, LeCouter J. The biology of VEGF and its receptors. Nat Med, 2003; 9(6), 669-676.
24. Naveed B, Comfort AL, Ferrier N, Segal LN, Kasturiarachchi KJ, Kwon S et al. Wtc dust induces GM-CSF in serum of fdny rescue workers with accelerated decline of lung function and in cultured alveolar macrophages. Am J Respir Crit Care Med, 2011; 183(1).
25. Pope CA, Bhatnagar A, McCracken JP, Abplanalp W, Conklin DJ, O'Toole T. Exposure to Fine Particulate Air Pollution Is Associated with Endothelial Injury and Systemic Inflammation. Circulation Research, 2016; 119(11), 1204-1214.
26. Niu J, Liberda EN, Qu S, Guo X, Li X, Zhang J et al. The role of metal components in the cardiovascular effects of PM2.5. PLoS One, 2013; 8(12).
27. Pelletier G, Rigden M, Kauri LM, Shutt R, Mahmud M, Cakmak S et al. Associations between urinary biomarkers of oxidative stress and air pollutants observed in a randomized crossover exposure to steel mill emissions. Int J Hyg Environ Health, 2017; 220(2 Pt B), 387-394.
28. Liu L, Ruddy T, Dalipaj M, Poon R, Szyszkowicz M, You H et al. Effects of indoor, outdoor, and personal exposure to particulate air pollution on cardiovascular physiology and systemic mediators in seniors. J Occup Environ Med, 2009; 51(9), 1088-1098.
29. Liu L, Urch B, Poon R, Szyszkowicz M, Speck M, Gold D et al. Effects of ambient coarse, fine, and ultrafine particles and their biological constituents on systemic biomarkers: a controlled human exposure study. Environ Health Perspect, 2015; 123(6), 534-540.
30. Higgins JP, Green S. Cochrane handbook for Systematic Reviews of In- terventions. John Wiley , Sons Ltd, Chichester UK, 2008; 243-293.
31. Higgins JP, Thompson SG, Deeks JJ, Altman DG. Measuring inconsis- tency in meta-analyses. BMJ, 2003; 327, 557e560.
32. Calderon-Garciduenas L, Rodriguez-Alcaraz A, Villarreal-Calderon A, Lyght O, Janszen D, Morgan KT. Nasal epithelium as a sentinel for airborne environmental pollution. Toxicol Sci, 1998; 46(2), 352-364.
33. Morishita R. Is vascular endothelial growth factor a missing link between hypertension and inflammation? Hypertension, 2004; 44(3), 253-254.
34. Zhao Q, Ishibashi M, Hiasa K, Tan C, Takeshita A, Egashira K. Essential role of vascular endothelial growth factor in angiotensin II-induced vascular inflammation and remodeling. Hypertension, 2004; 44(3), 264-270.
35. Brook RD, Bard RL, Kaplan MJ ,Yalavarthi S, Morishita M, Dvonch JT et al. The effect of acute exposure to coarse particulate matter air pollution in a rural location on circulating endothelial progenitor cells: results from a randomized controlled study. Inhal Toxicol, 2013; 25(10), 587-592.
36. Fang SC, Meht AJ, Alexeeff SE, Gryparis A, Coull B, Vokonas P et al. Residential black carbon exposure and circulating markers of systemic inflammation in elderly males: The normative aging study. Environ Health Perspect, 2012; 120(5), 674-680.
37. Iwanaga K, Elliott MS, Vedal S, Debley JS. Urban particulate matter induces pro-remodeling factors by airway epithelial cells from healthy and asthmatic children. Inhal Toxicol, 25(12), 2013; 653-660.
38. Xu X, Wang H, Liu S, Xing C, Liu Y. TP53-dependent autophagy links the ATR-CHEK1 axis activation to proinflammatory VEGFA production in human bronchial epithelial cells exposed to fine particulate matter (PM2.5). Autophagy, 2016; 12(10), 1832-1848.
39. Xu Xiu, Ao Deng its wood grid, Liu Sha Sha. PM2.5 Promotes Expression and Inflammatory Response of VEGF in Bronchial Epithelial Cells by Inducing AP-1 Activation . Military Medicine, 2016; 40 (7): 541-544. (In Chinese).
40. Liu Shasha, Ao Deng its wood grid, Wang Hongli. Expression of vascular endothelial growth factor in bronchial epithelial cells induced by activation of NF-κB pathway . Military Medicine, 2015; 5, 325-328. (In Chinese).
